# Blanching of Two Commercial Norwegian Brown Algae for Reduction of Iodine and Other Compounds of Importance for Food Safety and Quality †

**DOI:** 10.3390/foods14234113

**Published:** 2025-12-01

**Authors:** Maria Stavnes Sletta, Cecilie Bay Wirenfeldt, Maren Sæther, Øystein Arlov, Petra Ložnjak Švarc, Synnøve Strand Jacobsen, Finn Lillelund Aachmann, Håvard Sletta, Susan Løvstad Holdt, Inga Marie Aasen, Turid Rustad

**Affiliations:** 1 Department of Biotechnology and Food Science, Norwegian University of Science and Technology (NTNU), Sem Saelands vei 6/8, 7491 Trondheim, Norway; maria.stavnes.sletta@fkf.no (M.S.S.); wirenfeldtc@gmail.com (C.B.W.); synnove.s.jacobsen@ntnu.no (S.S.J.); finn.l.aachmann@ntnu.no (F.L.A.); 2 National Food Institute, Technical University of Denmark (DTU), Kemitorvet, 2800 Kongens Lyngby, Denmark; ploznjak@hotmail.com (P.L.Š.); suho@food.dtu.dk (S.L.H.); 3 Seaweed Solutions AS, Bynesveien 50C, 7018 Trondheim, Norway; sather@seaweedsolutions.no; 4 Department Biotechnology and Nanomedicine, SINTEF Industry, Richard Birkelands vei 3b, 7034 Trondheim, Norway; oystein.arlov@sintef.no (Ø.A.); havard.sletta@sintef.no (H.S.);

**Keywords:** seaweed, *Saccharina latissima*, *Alaria esculenta*, nutrient retention, chemical composition, minerals, vitamins

## Abstract

Two commercially utilized kelp species, winged kelp (*Alaria esculenta*) and sugar kelp (*Saccharina latissima*), can accumulate high amounts of iodine and thereby pose a health concern if consumed in excess. Water blanching is used industrially to reduce the iodine content. This study aimed to optimize the blanching conditions to reduce the energy consumption and environmental impact by investigating the parameters of temperature, duration, use of sea or fresh water, biomass-to-water ratio, and recycling of water. The study investigated the impact of the blanching conditions on composition of the biomass, including nutrient content and potential toxic elements. The iodine content was reduced to 5% of the initial content for *S. latissima* and to 8% for *A. esculenta* at the optimal conditions in the present study, which was blanching in seawater at 80 °C for 2 min. Using tap water at the same conditions resulted in a reduction to 7 and 11% of the initial content. The content of arsenic in blanched winged kelp was reduced to levels below the maximum allowed content in feed, whereas the content in blanched sugar kelp remained above this level. This study provides a comprehensive set of data on blanching of the two kelp species, with high relevance for the industrial scale-up of kelp processing.

## 1. Introduction

The cultivation and use of seaweed for foods have increased in the last decade in several European countries. The subtidal cold-water brown algae *Alaria esculenta* (winged kelp) and *Saccharina latissima* (sugar kelp) are the main cultivated species in Europe. The long coastline of Norway, with cold and clean waters, is ideal for kelp cultivation, and the number of cultivation sites, companies involved, and production output in Norway have grown substantially over the past decade. In 2023, the number of cultivation licenses was 522, compared to 54 in 2014 [1].

Kelp is the most effective accumulator of iodine of any living species [2]. Iodine is an essential trace element, and according to FAO/WHO, the recommended intake for adults is 150 µg day^−1^ [3], with a European specified upper intake level of 600 µg day^−1^ [4]. The iodine content of kelp varies depending on the species and the external iodine concentration of the seawater and environmental stressors [5]. Sugar kelp has the highest iodine content of the two kelps in this study, ranging from 3124 to 6568 mg (kg dw)^−1^, whereas winged kelp has concentrations from 213 to 945 mg (kg dw)^−1^ [6,7,8,9,10]. When included in the diet, the high iodine content in kelp may pose a risk of overconsumption, with adverse health effects [11]. Through a daily consumption of only 1.2 g of fresh, non-processed sugar kelp containing 0.5 mg iodine/g wet weight (approx. 5000 mg (kg dw^−1^)), consumers would reach the upper intake level [7].

Other potential hazards from the consumption of kelp are the potentially toxic elements, such as arsenic and cadmium [11,12]. Brown algae take up inorganic arsenic, presumably because it resembles the phosphate ion and converts it to organic compounds, which are predominantly arsenosugars [12]. Total arsenic is the commonly reported form of arsenic, but only the inorganic forms are considered toxic. Most kelp species have been shown to have a low inorganic arsenic-to-total arsenic ratio, with the inorganic form constituting less than 10% of the total content in sugar kelp and winged kelp [13], although this also depends on the levels and types of arsenic in their environments. Cadmium and other divalent potentially toxic elements (Pb^2+^, Cu^2+^, Ba^2+^) can accumulate in the biomass by binding to alginate through replacement of calcium in cross-linking junction zones [14].

Blanching is a common vegetable processing method and is used prior to freezing, drying, or canning to increase product quality by inactivating endogenous enzymes, decreasing microbial load, and removing potentially toxic constituents [15,16]. In conventional hot water blanching, the product is submerged in 70 to 100 °C water for several minutes, followed by draining and cooling. Blanching is well known to cause loss of nutrients and flavor compounds from the product, particularly water-soluble compounds such as carbohydrates, proteins, amino acids, minerals, and vitamins that are not bound in macromolecular or cellular structures [16]. Several studies have shown that blanching or boiling for 1–15 min is effective at reducing the iodine content of sugar kelp [7,17,18,19,20,21], and blanching has become the most frequently used method for iodine reduction in brown algae. This method has been implemented by some producers of cultivated brown algae for the food market, using a temperature of 80 °C for 2 min. These conditions have been found to reduce the iodine by 80–90% in sugar kelp [7]. However, very few studies on the effects of blanching fresh winged kelp for iodine reduction have been published to date. Of note, Nitschke and Stengel [20] showed that rehydrating and boiling in deionized water for 20 min reduced the iodine content of winged kelp from 670 to 165 mg (kg dw)^−1^. A deeper understanding of the impact of different blanching conditions is thus needed, including the development of more sustainable and less energy-demanding methods.

The main objective of this study was to optimize the blanching conditions to maximize the iodine reduction of the two commercial kelp species, sugar kelp and winged kelp, with emphasis on parameters that can improve logistics and reduce costs, such as using seawater for blanching, using less water, and/or blanching at lower temperatures. Blanching conditions that have previously been found to reduce iodine by 80–90% in sugar kelp [7] were chosen as a reference. This previous study was limited to sugar kelp and the measurement of iodine, whereas the present study also encompassed winged kelp and the impact of blanching conditions on potentially toxic elements, other minerals, and selected nutrients. For winged kelp, having a lower native iodine content, a central research question was whether iodine would be reduced in the same relative ratio as for sugar kelp or if the resulting content in blanched sugar kelp (200–300 mg (kg dw)^−1^) was the minimum level.

## 2. Materials and Methods

### 2.1. Materials

Commercially cultivated winged kelp and sugar kelp were harvested from the sea farm of Seaweed Solutions AS near Frøya, Norway (N 63°42.279′ E 8°52.232′) in May 2020. The cultivation site is classified as being open exposed coast, with euhaline (>30 PSU) water. After harvest, the seaweed was stored in tanks in membrane-filtered, UV-treated seawater (8 °C). The experiments were conducted on the same harvest batch over a span of two to three days. Stipes and holdfast were kept. The seaweeds had no visible fouling organisms. Winged kelp varied in length from 15 cm to 150 cm, with an average of 67 cm (*n* = 10), while sugar kelp varied from 10 cm to 100 cm, with an average of 52 cm (*n* = 10). The experiments (Exp.) started the same day that the seaweed was harvested (Day 1). For *A. esculenta*, the experiments were performed within two days after harvest (Day 1–2). Exp. 1 was run partly on Day 1 and partly on Day 2, while the remaining experiments were run on Day 2. For *S. latissima*, Exp. 1 was run on Day 1, Exp. 3 and Exp. 4 on Day 2, and Exp. 2 and Exp. 5 on Day 3.

All chemicals used were of analytical or chromatography grade.

### 2.2. Blanching Methods and Conditions

An overview of the experimental design is given in Table 1. The blanching was carried out in a water bath (20 L) with temperature control. The standard conditions used as reference were selected based on data from preliminary experiments and were as follows: whole seaweed blanched at 80 °C for 120 s, using fresh water for both blanching and cooling (FW, FW), at a ratio of 50 g wet seaweed per liter of water (50 g/L), without reusing the water (Rep 1).

In each of the experiments, one or more of the standard variables were replaced to evaluate its effect. Exp. 1 explored temperatures (45 and 80 °C) and durations (30 and 120 s). Exp. 2 investigated the ratio of seaweed to water. In Exp. 3, the seaweed was cut mechanically into pieces of 1–10 cm in width/length prior to blanching, denoted as “cut” in Table 1. This experiment was performed only for sugar kelp. In Exp. 4, the blanching and cooling liquid varied between fresh water (FW) and membrane-filtered, UV-treated seawater (SW). The final experiment, Exp. 5, examined the effect of blanching multiple times in the same water, thus reusing the water. For Exp. 1, 3, 4, and 5, samples of 500 g seaweed were blanched, whereas in Exp. 2 samples of 2.5 kg were blanched. The number of sporophytes in 500 g was at least 10. All blanching experiments were carried out in three replicates for each condition within the experiments, except for Exp. 2 and Exp. 5 for winged kelp, where only two replicates were included.

The blanching was carried out as follows: Fresh seaweed stored in filtered seawater was collected and left on a grate to drip for one minute. The seaweed was moved around while dripping to ensure removal of excess water from the surface. After dripping, 500 ± 10 g (2500 g in Exp. 2) of seaweed was weighed for blanching. The seaweed was soaked in a water bath containing 10 L (5 L in Exp. 2) pre-heated water for 30 or 120 s according to the experimental set-up. After blanching, the seaweed was directly transferred into a cooling bath. After one minute of cooling, the dripping procedure was repeated, and the blanched seaweed was weighed. The blanching water was sampled (40 mL) twice for each replicate in all blanching experiments, prior to collection of the seaweed samples for analysis. The samples were stored at −20 °C at the processing site for up to two weeks prior to being transported to the laboratory, where they were stored at −40 °C, or −80 °C for vitamin analyses.

### 2.3. Chemical Analysis

#### 2.3.1. Sample Preparation

The frozen seaweed samples, except those for vitamin analyses, were dried in a freeze-dryer (Alpha 1–4 LD plus, Christ, Osterode am Harz, Germany) at −60 °C and 0.1 mbar vacuum, before being coarsely ground using a food processor. The samples for mineral analyses were further milled (MM 400, Retsch, Haan, Germany, Settings: 30 Hz for 40 s). For vitamin analysis, the frozen seaweed at −80 °C was homogenized by a coffee grinder (Rommelsbacher EGK 200, Dinkelsbühl, Germany) using liquid nitrogen to keep it frozen.

All experimental replicates were analyzed as described below, using three analytical replicates if otherwise not stated.

#### 2.3.2. Dry Matter and Ash

The dry matter and ash content were determined gravimetrically on fresh and blanched seaweed (*n* = 2) according to AOAC Method 950.46 [22].

#### 2.3.3. Iodine, Inorganic Arsenic, and Other Minerals and Trace Elements

Minerals and trace elements were analyzed by ICP-MS, with sample preparation and analysis as described by Arlov et al. [10]. In short, the samples were extracted with 5 mL 20% (*v*/*v*) TMAH at 80 °C for analysis of the halogens (Cl, Br, I) and digested in 5% HNO_3_ for analysis of the other elements (Na, Mg, P, S, K, Ca, Fe, Co, Zn, As, Se, Cd, Ba). The prepared samples were analyzed on an Agilent 8800 Triple Quadrupole ICP-MS (Agilent Technologies, Santa Clara, CA, USA) (*n* = 4). Inorganic arsenic was quantified in a selection of the samples based on the methodology published by the National Institute of Standards and Technology [23], using KI/I2 to oxidize all inorganic As to As (V).

#### 2.3.4. Lipids

The gravimetric method described by Bligh and Dyer [24] was used to quantify total lipid content. Samples were analyzed in duplicate.

#### 2.3.5. Free and Total Amino Acids

For analysis of free amino acids, extracts were prepared as described by Stévant et al. [25] (*n* = 2). The samples were analyzed using a reversed-phase Ultra High Liquid Chromatography (RP-HPLC) (Dionex UltiMate^®^ 3000 UHPLC+ Focused, Dionex UltiMate^®^ 3000 Autosampler, Dionex RF Fluorescence Detector, Thermo Scientific, Waltham, MA, USA) and Novapak column (Nova-Pak C18 4 μm, 3.9 × 150 mm, Waters tech. Ltd., Wexford, Ireland). Protein precipitation was performed as described by Osnes and Mohr [26]. The quantifications are described by Nielsen et al. [7]. The extraction and quantification of total amino acids (excluding tryptophan) was done as described by Nielsen et al. [7] (*n* = 2). The chromatographic peaks of glycine and arginine were merged, and an average of their molar masses was used for calculation. The total protein content was calculated by summing the amino acids and then subtracting the water (18 g H_2_O (mol amino acid)^−1^), which was incorporated during hydrolysis.

#### 2.3.6. Carbohydrates

Carbohydrates were determined as monosaccharides after acid hydrolysis, followed by high-performance anion-exchange chromatography with pulsed amperometric detection (HPAEC-PAD), as described by Arlov et al. [10]. For calculation of polysaccharide mass, the added water during hydrolysis was subtracted. Three analytical replicates were used.

#### 2.3.7. Water-Soluble Vitamins (Vitamin C and Folate)

Vitamin C was quantified by the HPLC-UV method previously described by Wirenfeldt et al. [27] (*n* = 1). Folate was analyzed by LC-MS/MS using a single-enzyme extraction step as described by Ložnjak Švarc et al. [28]. Folate vitamers (tetrahydrofolate, 5-methyltetrahydrofolate, formyl forms and folic acid) were determined and expressed as folic acid equivalents, and their sum was reported as total folate content [28]. One of the experimental replicates was analyzed with three analytical replicates.

### 2.4. Microbial Counts

Total aerobic viable count was determined as colony forming units (CFU) by the NMKL Method 184 [29] on Compact Dry TC plates (Labolytic, Trondheim, Norway). Briefly, one sample of 5 g of seaweed from each of the experimental replicates and 45 g of peptone saline (1 g peptone and 8.5 g NaCl in 1 L deionized water, autoclaved) were mixed for one minute in a stomacher bag. One mL from either the stomacher bag or a tenfold dilution series was inoculated on the Compact Dry TC and incubated at room temperature for 3–4 days. CFU were counted and reported as log CFU per g wet seaweed.

### 2.5. Data Presentation and Statistics

All data are presented as mean ± standard deviation unless otherwise stated. One way Analysis of Variance (ANOVA) was conducted in R Studio software version 1.4.1106 with the treatments as factors [30]. A significance level of *p* < 0.05 was used to assess statistical significance. Homogeneity of variance across groups was confirmed by Levene’s test. The pair-wise comparison information was acquired using Tukey’s Honest Significant Difference (HSD). Pearson correlation was performed to reveal correlations between minerals and trace elements. Principal Components Analysis (PCA) was performed using R Studio version 1.4.1106 on a standardized and scaled data matrix for minerals and trace elements. Samples not included in the ANOVA are winged kelp 500 g/L and Reps. 5 and 10.

## 3. Results and Discussion

### 3.1. Proximate Composition of Fresh and Blanched Biomass

**Figure 1:**
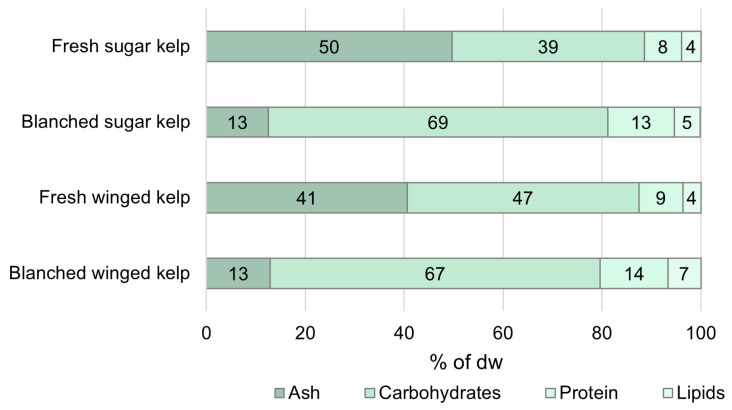
Effect of blanching on the composition of sugar kelp and winged kelp. The blanched seaweeds were treated in standard conditions: 80 °C for 2 min. All data are presented as the average in % of dw; the carbohydrates were calculated by subtracting the other proximates from 100%.

As illustrated in Figure 1, the composition of the two species was different. Sugar kelp had a higher ash content, and since the protein and lipid contents were similar, the calculated carbohydrate content was lower. However, after blanching under standard conditions, the two kelps had similar proximate compositions. Blanching and cooling in tap water reduced the dry weights for both species, from 11.0 to 7.2–9.9% for winged kelp, and from 8.6 to 4.5–6.7% for sugar kelp, as shown in Table 2, which includes data for all blanching conditions. This loss was caused by the concentration gradients, resulting in an efflux of soluble components. With washing and/or cooling in seawater, the dry weights were not significantly changed, due to the smaller differences in osmotic pressure. For both species, the protein content in the SW, SW blanched kelp was maintained at the same concentration as the fresh kelp, while those blanched or cooled in tap water all had significantly higher protein contents than the fresh kelp (ANOVA; *p*< 0.001). This can be explained by the loss of other components when using tap water as well as the insolubility of most of the protein (only free amino acids, peptides, and soluble protein were lost).

### 3.2. Reduction of Minerals and Trace Elements by Blanching at Different Conditions

**Figure 2:**
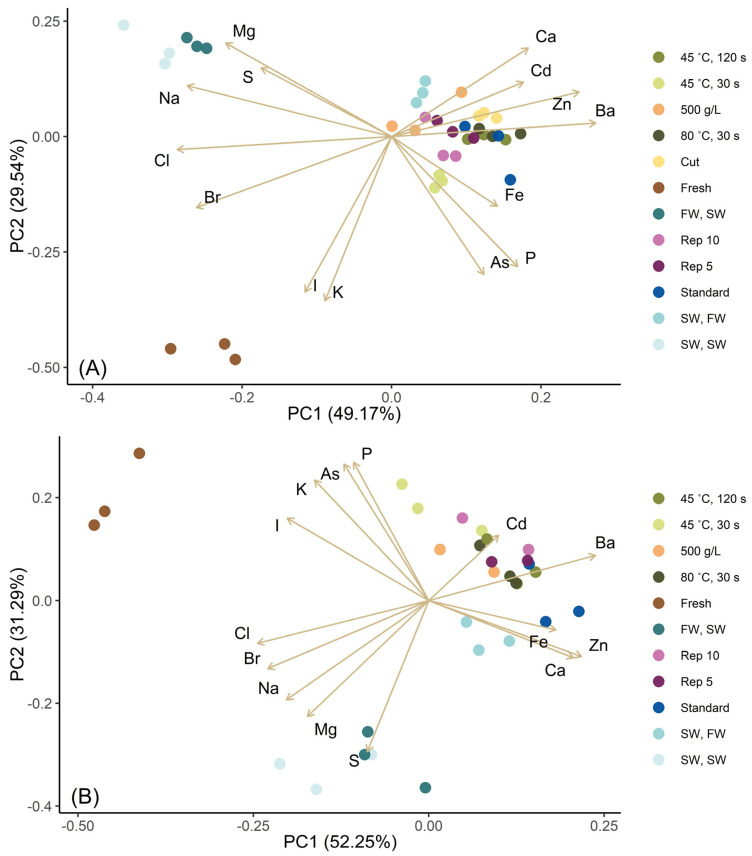
Bi-plot with PCA scores and loadings for minerals and trace elements of sugar kelp (**A**) and winged kelp (**B**) blanched under different conditions (colors). Each data point indicates one experimental replicate, with a total of 3 experimental replicates for each treatment type, except for (**B**) 500 g/L, Rep 10 and Rep 5, with only two experimental replicates. A total of 78.7% (**A**) and 83.5% (**B**) of the variance is explained by PC1 and PC2.

For more detailed data on the effect of the blanching conditions on the reduction in ash content, in total 16 minerals and trace elements were analyzed, including iodine and the potential toxic elements arsenic and cadmium (Table 3 and Supplementary Figure S1). Their correlation has been visualized in bi-plots (Figure 2) and heat maps (Supplementary Figure S3), which illustrate the effects of the different blanching conditions. Potassium (K) and I were the only two elements that were reduced by all the blanching treatments, indicating that the major share of K and I are not bound to insoluble biomass components and that there was no influx of these ions from seawater when seawater was used for cooling. A positive linear correlation was observed between K and I for both species, with a Pearson’s correlation coefficient higher than 0.9 (Supplementary Figure S4). Usually, 80–90% of iodine in kelp is stored as the inorganic form iodide (I^−^) [19]. The most likely explanation for the correlation is that the anion iodide needs a positive counter cation to efflux simultaneously, where K^+^ is the dominating cation in the biomass, constituting 14% of the dry weight and 29% of the ash for sugar kelp.

Most treatments, with the exception of SW, SW and FW, SW, which are discussed below, also showed a reduction of arsenic (As), bromine (Br), chlorine (Cl), magnesium (Mg), sodium (Na), phosphorus (P), and sulfur (S). Pearson’s correlation (Supplementary Figure S3) showed that cadmium (Cd) and iron (Fe) did not correlate with any other minerals or trace elements in either species. This is also partly illustrated in the bi-plots (Figure 2), as the loadings (arrows) of these specific minerals are not in the same dimensions as the others.

Blanched biomass from the two treatments FW, SW and SW, SW, which involved cooling in seawater, not only had a higher ash content (Table 2) but also an different mineral profile (Table 3) than those cooled in tap water (FW, FW and SW, FW). This indicates an influx of ions from the seawater. It is noteworthy that the two halogens Cl and Br were retained in the kelp with SW, SW and FW, SW blanching, when these two methods were in fact among the most efficient to reduce the other halogen, iodine (Table 3, Supplementary Figure S2). The data points of these two treatments (SW, SW and FW, SW) followed the loadings of Br, Cl, Mg, Na, and S. The fluxes in and out of the biomass will, unless the ions are associated with retained macromolecules, be governed by the concentration gradients. When blanching in fresh water and cooling in seawater, the concentration gradients will imply a considerable influx of ions from the seawater, resulting in a mineral composition, resembling seawater. When blanching and cooling in seawater, the differences in osmotic pressure will be small, with minimal fluxes in and out of the biomass. However, an equilibration of the concentrations of individual minerals with time is likely to occur. Seawater has a near twofold concentration of sodium compared to kelp (wet weight) and a high concentration of S, as sulfate [31], explaining the retention of these ions and the exchange of K with Na.

### 3.3. Iodine Reduction, Blanching Water Recycling, and Allowable Seaweed Consumption Levels

Currently, no regulations exist for the maximum iodine content allowed in food. Only the French Food Safety Agency (AFSSA) has recommended a maximum content of 2000 mg iodine (kg dw)^−1^ [32]. The initial iodine contents in the fresh, unprocessed seaweed used in this study were 4818 ± 331 and 682 ± 95 mg (kg dw)^−1^ in sugar kelp and winged kelp, respectively, meaning that the level in sugar kelp was more than twice the AFSSA recommendation. The iodine levels were within the range of previous analyses of biomass from the same geographic region [8,10].

**Figure 3:**
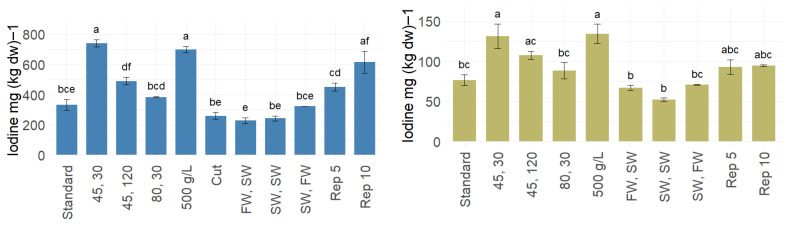
Iodine levels (mg (kg dw)^−1^) in sugar kelp (**left**) and winged kelp (**right**) after blanching under different conditions (Table 1). Means are presented as bars, and standard deviation as error bars. Significant difference (ANOVA; *p* < 0.05) is illustrated by lower-case letters (a–f) within each sub-graph.

In the present study, the blanching conditions presented in Table 1 reduced the iodine levels to 228–741 for sugar kelp and 52.1–134 mg (kg dw)^−1^ for winged kelp, corresponding to an 85–95% and 80–92% reduction for sugar kelp and winged kelp, respectively (Table 3). This confirms that iodine can be reduced in the same relative ratio for winged kelp as for sugar kelp. The different blanching conditions led to significantly different levels of iodine, as illustrated in Figure 3 (ANOVA; *p* < 0.001, F = 36.4). The maximum iodine reduction was achieved by treatments at 80 °C. For both species, all the 80 °C treatments reached similar levels of iodine after blanching, except for sugar kelp blanched by reusing the water (Reps 5 and 10) and the increased seaweed-to-water ratio (500 g L^−1^). The reduction obtained at 80 °C was the same as previously obtained by Nielsen et al. [7] for sugar kelp. Both studies showed that at this temperature, the blanching duration did not have an influence. Blanching at 45 °C was investigated to see if low temperatures could give sufficient iodine reduction. This temperature resulted in an iodine content of 15.5% of the initial value using 30 s, and 10.4% after 2 min. Using membrane-filtered, UV-treated seawater (SW, FW and SW, SW) and cutting the sugar kelp (Cut) gave the same iodine reduction as the standard conditions. At the optimum conditions, which were blanching in seawater at 80 °C for 2 min, the iodine contents were reduced to 5% (*S. latissima*) and 8% (*A. esculenta*) of their initial contents. Using tap water under the same conditions reduced the contents to 6.9 and 11.2% of the initial contents. Using seawater for blanching instead of fresh water has great potential, as it is easily accessible, has a lower cost, and is more environmentally sustainable for near-shore or offshore operations.

Increasing the seaweed-to-water ratio from 50 to 500 g L^−1^ resulted in less effective iodine reduction for both species. In addition, the water temperature dropped by approximately 20 °C when adding this larger proportion of seaweed, which presumably reduced the blanching efficiency, as the kelp took longer to reach the target temperature. The lower iodine efflux with increased seaweed-to-water ratio could also be due to iodine saturation in the water. Reusing blanching water is another option for reducing costs, water consumption, and carbon emissions. For winged kelp, the same water could be used at least ten times under standard conditions without a negative impact on iodine reduction. However, for sugar kelp, the blanching effect was substantially affected by the fifth (Rep 5) and tenth (Rep 10) repetitions with the concentration of 50 g kelp to 1 L blanching water (Figure 3). Thus, the iodine equilibrium between kelp and water appears to be reached when blanching sugar kelp five times in the same water. The differences in iodine reduction observed for the two species when reusing blanching water further supports that the iodine efflux depends on the concentration in the blanching water. By modelling with an assumption of linearity, it was possible to predict when the blanching water should be changed, when using standard conditions. As an example, for reduction of the iodine content of sugar kelp to the AFSSA recommendations of a maximum of 2000 µg/g, 2.7 tons biomass can be blanched in 1 m^3^ water, while for reduction to the same levels in winged kelp (approx. 600 µg/g), 480 kg can be blanched. However, as the iodine efflux may not follow a linear trend, it is recommended to study this further.

Based on the obtained results, blanching can increase the maximum acceptable intake of kelp from 0.125 g dry weight (1.44 g ww) to 0.81–2.6 g dry weight (9.3–48.3 g ww) for sugar kelp. For winged kelp, the corresponding amounts increased from 0.88 g dry weight (8.0 g ww) to 4.5–11.5 g dry weight (51–109 g ww) (Supplementary Figure S4).

### 3.4. The Possibility of Lowering the Potentially Toxic Elements, Arsenic and Cadmium, Using Various Blanching Conditions

No regulation on the maximum allowed levels of total arsenic in food products exists. For animal feed, the maximum level given by the EFSA is 40 mg kg^−1^ in seaweed meal with 12% moisture content [33], which is equivalent to 44.8 mg (kg dw)^−1^. For winged kelp, total arsenic was significantly reduced by the blanching (ANOVA; *p* < 0.001, F = 15.6). The initial concentration was 47.9 ± 2.7 mg (kg dw)^−1^, and the maximum level in blanched biomass was 37 mg (kg dw)^−1^, with an average of 28.5 ± 5.0 mg (kg dw)^−1^, which meant that all the studied blanching conditions resulted in total As levels below the EFSA level (Table 3 and Supplementary Figure S1). For sugar kelp, the blanching conditions of 500 g/L, FW, SW, and SW, SW led to a significant reduction of total arsenic (39.8 ± 0.1 mg (kg dw)^−1^) compared to fresh sugar kelp (66.7 ± 13.8 mg (kg dw)^−1^) (ANOVA; *p* < 0.001, F = 16.5). The other blanching methods (SW, FW, FW, FW) did not reduce the arsenic content compared to fresh sugar kelp and had an average of 58.1 ± 6.1 mg (kg dw)^−1^, which is above the maximum allowed levels in feed products. These results suggest that cooling with seawater after blanching is more efficient than fresh water in reducing the total arsenic levels on a dw basis, due to the lower loss of minerals and other soluble compounds and the maintenance of a higher dry weight. However, in other studies, significant reductions were obtained by blanching in fresh water, e.g., from 63 to 36 mg (kg dw)^−1^ at 92–99 °C for 15 min. in a ratio of 102 g kelp per L^−1^ fresh water, and from 72 to 54 mg (kg dw)^−1^ at 60 °C for 2 min [34]. Jönsson et al. [17] obtained a reduction from 62 to 34 mg (kg dw)^−1^ under conditions similar to the standard conditions used in the present study.

A recent legislative focus has been to monitor inorganic arsenic rather than total arsenic, as inorganic arsenic (arsenate and arsenite) is assumed to be more toxic than organic arsenic and is known to be carcinogenic [33]. The Norwegian regulation on animal feed set the maximum allowed level of inorganic arsenic in feed based on macroalgae to 2000 µg kg^−1^ [35]. Neither the fresh nor blanched winged kelp used in the present study had inorganic arsenic levels above the Level of Quantification (LOQ) of 33 µg (kg dw)^−1^, which is considerably lower than the 220 µg (kg dw)^−1^ inorganic arsenic level previously detected in French winged kelp [9]. The fresh sugar kelp in the present study contained 55.3 ± 14.6 µg (kg dw)^−1^, and the blanched biomass (standard conditions) contained 123 ± 48 µg (kg dw)^−1^. Because of the large standard variations, no significant differences were found by the ANOVA analysis. However, a significant reduction in inorganic arsenic from 214 to 30.3 µg (kg dw)^−1^ was obtained by Jönsson et al. [17] when sugar kelp was blanched at 80 °C for 2 min. When blanching with seawater, FW, SW had 68.5 µg (kg dw)^−1^, SW, FW had 97.8 µg (kg dw)^−1^, and SW, SW was below the LOQ. Thus, all samples analyzed contained less than 7% of the maximum allowed level according to Norwegian regulation. Despite the emphasis on inorganic arsenic, it should be noted that no information on the toxicity of organic arsenolipids and arsenosugars exists [33,36], but both are present in the two kelp species [37]. Therefore, it cannot be concluded whether the detected arsenic levels affect food safety or not.

In the present study, Cadmium content was not reduced by blanching in either kelp species (Table 3 and Supplementary Figure S1). Divalent cations bind to alginate, and due to the reduction of other compounds, the relative content actually increased in some cases. Fresh sugar kelp contained 0.838 ± 0.085 mg (kg dw)^−1^, and the average in blanched sugar kelp was 1.30 ± 0.20 mg (kg dw)^−1^ (ANOVA; *p* < 0.001, F = 5.63). Winged kelp contained more cadmium, with levels of 2.09 ± 0.05 mg (kg dw)^−1^ in fresh kelp and, on average, 2.45 ± 0.35 mg (kg dw)^−1^ in blanched biomass, with no significant differences (ANOVA; *p* = 0.103, F = 1.94). The values in fresh biomass were within the range reported by others [25,34,36]. Blikra et al. [33] did not obtain a reduction in Cd when boiling sugar kelp, and the content increased from 0.55 to 0.94 mg (kg dw)^−1^ in the study by Jônsson et al. [17]. The legislative threshold value of cadmium in seaweed used as a food supplement is 3 mg (kg dw)^−1^ [38]. In adults, average exposure to cadmium in Europe is already close to the tolerable weekly intake (TWI), with the main sources being rice, grains, and vegetables. However, Sá Monteiro et al. [36] reported that the concentrations of cadmium in Danish seaweed would, with a serving size of 5 g freeze-dried seaweed, contribute to only 1.2–3.5% of the TWI, which is marginal compared to other sources. However, if the serving size increases, kelp could be a source of undesirable cadmium intake.

### 3.5. Loss of Flavor Compounds and Nutrients

Kelps have a high content of free glutamic and aspartic acid, two amino acids known to contribute to the taste of umami [39]. Moving towards a more plant-based diet, a plant-based umami source is of high interest for food producers. For both species, a noteworthy loss of the two free “umami” amino acids occurred during blanching under standard conditions (Table 4). Hamid et al. [16] found a large decrease in the same amino acids in the kelp *Undaria pinnatifida* within 20 s of blanching at 100 °C. Since these amino acids are heat stable, the loss must be attributed to leaching from the biomass.

Vitamin C and folate are water-soluble vitamins labile to various processing conditions [40,41] and potentially can be lost during blanching. The fresh sugar kelp contained 3.1 mg (100 g ww)^−1^ vitamin C. This is lower than the content in a batch of Danish cultivated sugar kelp, which contained 8.73 mg vitamin C (100 g ww)^−1^ [27]. The vitamin C content was reduced to below the detection limit of the analytical method following blanching under standard conditions. For winged kelp, treatment at 45 °C for 30 s was also analyzed. Here, the vitamin C content was lowered to 0.573 ± 0.034 mg (100 g ww)^−1^, which was a decrease to 13% of the level in fresh biomass. The loss of vitamin C during blanching is consistent with previous studies [41] and proves that even short time blanching at 45–80 °C will lower the vitamin C content considerably. The recommended vitamin C intake given by the FAO/WHO is 45 mg day^−1^ [3], which means that blanched kelp would not contribute to the vitamin C intake.

Folate is a generic term for a group of vitamers found mainly in leafy vegetables, legumes, offal, certain fruits, and cereals [40]. The total folate content found in fresh winged kelp was 113 ± 37 µg (100 g ww)^−1^, which indicates that this kelp could be considered as a good folate source, considering the EFSA recommendations of 330 µg day^−1^ for adults [42]. The folate content of sugar kelp was considerably lower (18.3 ± 1.7 µg (100 g ww)^−1^). The most abundant folate vitamer in both kelps was 5-methyltetrahydrofolate (≥90%) (Supplementary Tables), followed by formyl forms, which agrees with the results of Rodríguez-Bernaldo De Quirós et al. [43], who studied folate in other types of seaweed. Folate is known to be sensitive to processing due to oxidation, leaching, or the thermal instability of vitamers [40]. Under the standard blanching conditions (80 °C, 120 s), the loss of folate was significant, at 52% and 75% on dw basis, for sugar kelp and winged kelp, respectively. Due to this loss, winged kelp processed by the studied conditions would not contribute considerably to the daily intake of folate.

As seen in Table 4, the observed loss for the studied vitamins and free amino acids during blanching was in the same range as for other small, water-soluble molecules, with K as an example, and can therefore be explained by a physical loss due to efflux to the blanching water. However, it is likely that the high temperature contributed to an additional loss of Vitamin C.

### 3.6. The Effect of Blanching on the Soluble Carbohydrates of the Biomass

The soluble carbohydrates in brown algae are mannitol, laminaran, and fucoidan. Mannitol is a sugar alcohol that acts as energy storage and an osmoprotectant [44], laminaran is a small β-1,3 glucan (Mw approx. 5 kDa) used for energy storage, and fucoidan is a large, sulfated polysaccharide located within the cell walls and intercellularly in brown algae and is believed to prevent algae from drying out [45]. These components are of less relevance for food safety or nutrition, but the two polysaccharides have demonstrated health-beneficial properties as ingredients in food or feed, either as part of the seaweed biomass or as isolated compounds. Understanding the release of these compounds during blanching is thus important, and their concentrations in the blanching water were analyzed (Table 5).

All three carbohydrates were easily released from the seaweed into the blanching water. The concentrations in the blanching water increased with a factor of approx. 5 and 11–14 after five (Rep 5) and ten (Rep 10) reuses of the blanching water, relative to blanching under standard conditions. Interestingly, by using seawater (SW, SW) for blanching, the loss of carbohydrates to the blanching water was significantly reduced.

Laminaran is only partially water-soluble at room temperature but fully dissolves above 50 °C [46]. This effect was evident from a relatively lower release of laminaran by blanching at 45 °C. Fucoidan is a large molecule, with a molecular mass above 100,000 Da. Despite the size, this molecule was released in approximately the same ratios as laminaran and mannitol, with one exception. Cutting of the biomass had a far more pronounced effect on the fucoidan release than on the release of the two other compounds. After blanching of the cut biomass, the fucose concentration in the blanching water was almost at the same level as after five repetitions for the whole biomass. This shows that opening the tissue structures leads to the solubilization of more fucoidan, while the two other compounds were already fully soluble. A key finding is thus that to retain the soluble carbohydrates, seawater should be used for blanching and cooling, while cutting should be avoided to reduce the leaching of fucoidan.

### 3.7. The Decrease in Microbial Load

The initial aerobic viable count (AVC) on fresh, unprocessed sugar kelp was 3.52 ± 0.20 log CFU g^−1^ and decreased significantly (ANOVA; *p* = 0.001; F = 8.34) to 1.80–2.32 log CFU g^−1^ under all of the blanching conditions analyzed (Standard; 45 °C, 30 s; 45 °C, 120 s; 80 °C, 30 s; SW, SW). Similar results were found for fresh winged kelp, which had 3.87 ± 0.02 log CFU g^−1^ and decreased significantly (ANOVA; *p* = 0.017; F = 5.05) to 1.50–2.40 log CFU g^−1^ for the methods analyzed (Standard; 45 °C, 30 s; 45 °C, 120 s; 80 °C, 30 s). The filtered seawater had 0.85 log CFU mL^−1^, and no AVC was detected in any heated blanching water.

A recent study on Danish cultivated sugar kelp found an initial AVC between 4.0 and 4.5 log CFU g^−1^, whereas blanching (80 °C, 120 s) lowered the AVC to 0.9–1.8 log CFU g^−1^ cultured on marine agar [27]. These findings are comparable to this study; however, Blikra et al., [47] found 1.1–2.0 log CFU g^−1^ in both raw and heat-treated sugar and winged kelp cultured on marine agar. It must be noted that the initial AVC will depend on the natural environment of the kelps.

The AVC decreased to below 2.40 log CFU g^−1^ when blanched under any conditions, including blanching at 45 °C. These results showed that the kelp processors can blanch at 45 °C and still reduce potential microbial hazards. Wirenfeldt and Sørensen et al. [27] suggest using 7 log CFU g^−1^ as a threshold of shelf-life; the AVC levels found in this study are all below that threshold.

## 4. Conclusions

This study confirmed that the iodine content of sugar kelp and winged kelp can be efficiently reduced by blanching and that the same relative decrease can be obtained for winged kelp as for sugar kelp. Furthermore, blanching in seawater at standard conditions was as efficient as in tap water. Reuse of the blanching water up to ten times did not reduce the efficiency for winged kelp, but a significant reduction was seen for sugar kelp. Increasing the seaweed-to-water ratio limited the iodine reduction for both species. Blanching at optimal conditions increased the amount of kelp that can be consumed daily before exceeding the maximum recommended iodine intake of 600 µg/day from 0.125 to 2.6 g dw for sugar kelp and from 0.88 to 11.5 g dw for winged kelp.

Blanching and cooling in tap water led to the loss of bromine, chlorine, magnesium, sodium, and potassium, while cooling in seawater decreased arsenic, potassium, and phosphorus. Total arsenic content in blanched winged kelp was below maximum allowed levels in feed. In most of the blanched sugar kelp, As contents were above allowed levels in feed; however, the content of inorganic As was lower than 7% of the allowed levels. The cadmium content was below a European legislative threshold value for feed. Free amino acids, water-soluble vitamins, and water-soluble carbohydrates were lost to the blanching water when blanching in tap water, while blanching with seawater retained the carbohydrates.

Overall, the results showed that blanching in seawater at 80 °C can be well suited for the industrial processing of kelp, as it reduces iodine and arsenic levels, inactivates microorganisms, and retains valuable carbohydrates.

## Figures and Tables

**Table 1:** Summary of the different blanching conditions and the abbreviations of the experiments. Conditions deviating from the standard are shown in bold.

Set-Up	Abbreviation for Condition	Seaweed-to-Water Ratio (g ww) L^−1^	Blade	Blanching Water, Cooling Water	Temp (°C), Duration (s)	Number of Blanchings in Same Water	Reuse of Water	Experimental Replicates	
								Sugar Kelp	Winged Kelp
Standard	Standard	50	whole	FW, FW	80, 120	1	No	*n* = 3	*n* = 3
Exp. 1	45 °C, 30 s 45 °C, 120 s 80 °C, 30 s	50	whole	FW, FW	**45, 30** **45, 120** **80, 30**	1	No	*n* = 3 *n* = 3 *n* = 3	*n* = 3 *n* = 3 *n* = 3
Exp. 2	500 g/L	**500**	whole	FW, FW	80, 120	1	No	*n* = 3	*n* = 2
Exp. 3	Cut	50	**cut**	FW, FW	80, 120	1	No	*n* = 3	-
Exp. 4	FW, SW SW, SW SW, FW	50	whole	**FW, SW** **SW, SW** **SW, FW**	80, 120	1	No	*n* = 3 *n* = 3 *n* = 3	*n* = 3 *n* = 3 *n* = 3
Exp. 5	Rep X	50	whole	FW, FW	80, 120	**1–10**	**Yes, up to 9 times**	*n* = 3	*n* = 2

Exp. 2 and Exp. 5 were performed in duplicate (*n* = 2) for winged kelp; all other experiments were performed in triplicate (*n* = 3). Rep: Repeated blanching in the same blanching water. X in “Rep X” denotes number of blanchings in the same water (Rep 10 equals 10 sequential blanchings in the same water). FW: Fresh water; SW: Seawater.

**Table 2:** Composition of sugar kelp and winged kelp after blanching at different conditions, as described in Table 1. All values are given as avg ± std dev.

Blanching Conditions	*S. latissima*			*A. esculenta*		
	Dry Weight [% of ww]	Ash [% of dw]	Protein [% of dw]	Dry Weight [% of ww]	Ash [% of dw]	Protein [% of dw]
Standard	4.5 ± 1.2	12.5 ± 1.3	13.4 ± 1.2	7.2 ± 0.84	12.9 ± 0.46	13.7 ± 1.5
45 °C, 30 s	6.6 ± 0.84	16.5 ± 1.1	12.0 ± 0.05	8.9 ± 1.2	18.7 ± 3.5	11.5 ± 0.93
45 °C, 120 s	5.2 ± 1.1	13.3 ± 1.3	14.3 ± 0.19	9.9 ± 1.3	16.4 ± 2.8	12.4 ± 1.1
80°, 30 s	5.3 ± 0.75	13.9 ± 1.7	13.1 ± 0.55	9.9 ± 2.1	13.8 ± 1.2	12.3 ± 1.6
FW, SW	8.0 ± 0.63	39.9 ± 2.9	-	11.7 ± 1.1	28.1 ± 2.1	-
SW, SW	9.6 ± 1.4	41.9 ± 3.7	7.2 ± 0.33	13.3 ± 1.4	31.8 ± 3.7	8.8 ± 0.44
SW, FW	6.7 ± 1.0	17.8 ± 3.2	-	10.8 ± 1.6	16.3 ± 1.7	13.2 ± 0.54

**Table 3:** Content of minerals in fresh biomass and after blanching at different conditions (Table 1). All values are given as average ± std dev.

	**Blanching Conditions—*S. latissima***							
**Minerals**	**Fresh**	**Standard**	**45 °C, 30 s**	**45 °C, 120 s**	**80 °C, 30 s**	**FW, SW**	**SW, SW**	**SW, FW**
I [mg/kg dw]	4818 ± 405	331 ± 61	741 ± 41	490 ± 44	384 ± 8	228 ± 34	243 ± 28	324 ± 2
As [mg/kg dw]	67 ± 13	63 ± 4.0	68 ± 2.2	60 ± 2.5	65 ± 1.5	40 ± 1.6	40 ± 4.3	54 ± 4.5
Cd [mg/kg dw]	0.8 ± 0.25	1.3 ± 0.14	1.1 ± 0.08	1.2 ± 0.11	1.4 ± 0.05	1.1 ± 0.11	1.0 ± 0.08	1.6 ± 0.10
Br [mg/kg dw]	1768 ± 360	297 ± 27	593 ± 17	428 ± 16	383 ± 1	1072 ± 37	1173 ± 39	628 ± 25
Na [g/kg dw]	53 ± 1.8	11 ± 1.7	11 ± 0.95	10 ± 0.20	11 ± 0.89	103 ± 5.1	111 ± 11	40 ± 0.85
K [g/kg dw]	143 ± 50	30 ± 9.7	50 ± 4.1	34 ± 3.0	34 ± 2.3	18 ± 3.2	18 ± 2.6	17 ± 1.9
Cl [g/kg dw]	232 ± 50	12 ± 9.3	32 ± 4.0	11 ± 1.4	15 ± 2.2	219 ± 11	240 ± 19	68 ± 4.9
Ca [g/kg dw]	9.1 ± 0.12	13 ± 0.77	12 ± 0.42	14 ± 0.17	14 ± 0.56	12 ± 0.71	12 ± 0.38	14 ± 0.70
Mg [g/kg dw]	8.5 ± 0.23	8.5 ± 0.48	7.1 ± 0.36	8.3 ± 0.29	8.7 ± 0.49	15 ± 0.68	17 ± 1.2	12 ± 0.11
P [g/kg dw]	3.2 ± 0.65	3 ± 0.19	2.8 ± 0.11	2.9 ± 0.07	3 ± 0.11	1.8 ± 0.09	1.8 ± 0.16	2.7 ± 0.09
S [g/kg dw]	8.4 ± 0.59	9.3 ± 0.62	9.9 ± 0.42	11 ± 0.20	10 ± 0.53	12 ± 0.52	13 ± 0.99	11 ± 0.23
Fe [mg/kg dw]	56 ± 1.4	100 ± 41	85 ± 5.7	106 ± 11	76 ± 5.5	40 ± 4.0	52 ± 9.3	92 ± 17
Zn [mg/kg dw]	26 ± 4.6	45 ± 6.7	42 ± 3.5	47 ± 3.1	53 ± 4.3	35 ± 2.5	32 ± 2.9	50 ± 2.9
	**Blanching Conditions—*A. esculenta***							
**Minerals**	**Fresh**	**Standard**	**45 °C, 30 s**	**45 °C, 120 s**	**80 °C, 30 s**	**FW, SW**	**SW, SW**	**SW, FW**
I [mg/kg dw]	682 ± 120	76 ± 12	131 ± 26	108 ± 9.0	88 ± 18	67 ± 5.8	52 ± 3.5	71 ± 1.1
As [mg/kg dw]	48 ± 3.3	27 ± 0.96	33 ± 2.4	28 ± 1.9	31 ± 2.3	21 ± 0.95	20 ± 1.0	27 ± 1.4
Cd [mg/kg dw]	2.1 ± 0.05	2.3 ± 0.49	2.7 ± 0.10	2.8 ± 0.45	2.2 ± 0.18	1.8 ± 0.14	2.2 ± 0.76	2.6 ± 0.12
Br [mg/kg dw]	886 ± 110	117 ± 9.3	195 ± 25	164 ± 25	147 ± 38	657 ± 49	741 ± 54	256 ± 25
Na [g/kg dw]	77 ± 9.3	8.3 ± 1.7	14 ± 2.3	11 ± 0.37	12 ± 2.2	76 ± 2.7	88 ± 3.6	33 ± 1.9
K [g/kg dw]	94 ± 4.0	20 ± 6.1	60 ± 13	38 ± 5.4	36 ± 6.5	14 ± 4.8	13 ± 1.8	15 ± 3.7
Cl [g/kg dw]	251 ± 34	11 ± 5.1	62 ± 13	31 ± 6.5	31 ± 9.9	145 ± 5.9	174 ± 18	52 ± 6.2
Ca [g/kg dw]	10 ± 0.78	14 ± 0.41	12 ± 0.28	14 ± 0.76	13 ± 0.52	13 ± 0.51	12 ± 0.83	13 ± 0.30
Mg [g/kg dw]	12 ± 0.96	8.7 ± 0.38	7.4 ± 0.10	7.9 ± 0.27	8.1 ± 0.32	12 ± 0.06	13 ± 0.49	10 ± 0.23
P [g/kg dw]	4.3 ± 0.28	3.0 ± 0.07	3.1 ± 0.31	3.0 ± 0.13	3.1 ± 0.07	2.3 ± 0.10	2.2 ± 0.11	3.0 ± 0.09
S [g/kg dw]	9.4 ± 0.29	8.8 ± 0.69	8.1 ± 0.53	9.0 ± 0.28	8.9 ± 0.22	12 ± 0.45	12 ± 0.55	9.7 ± 0.17
Fe [mg/kg dw]	95 ± 15	147 ± 24	122 ± 9.3	132 ± 18	140 ± 2.5	139 ± 19	106 ± 19	140 ± 13
Zn [mg/kg dw]	39 ± 2.4	80 ± 11	60 ± 9.7	72 ± 5.6	74 ± 3.7	72 ± 12	64 ± 7.2	80 ± 6.3

**Table 4:** The content of the valuable nutrients and flavor compounds: vitamin C, total folate, free aspartic acid, and free glutamic acid, in the two kelp species before and after blanching. The corresponding values for potassium are included for comparison. Values are given as average ± std dev.

	Vitamin C ^(1);^	Total Folate ^(1)^	Free Aspartic Acid	Free Glutamic Acid	Potassium
	mg (g dw)^−1^	µg (g dw)^−1^	mg (g dw)^−1^	mg (g dw)^−1^	mg (g dw)^−1^
Sugar kelp					
Fresh	0.37 ± 0.04	2.19 ± 0.20	1.68 ± 0.49	1.16 ± 0.40	143 ± 50
Standard blanched	<LOQ	1.05 ± 0.014	0.261 ± 0.105	0.334 ± 0.169	30.0 ± 9.7
Loss [%]	-	52	84	71	79
Winged kelp					
Fresh	0.35 ± 0.03	9.27 ± 3.8	1.02 ± 0.35	1.14 ± 0.12	93.9 ± 4.0
Standard blanched	<LOQ	2.31 ± 1.09	0.142 ± 0.094	0.359 ± 0.137	19.8 ± 6.1
Loss [%]	-	75	86	68	79

(1) Vitamin C and folate were analyzed in wet biomass (values in Supplementary Tables). The values in the table are recalculated to dw for comparison.

**Table 5:** Carbohydrates in fresh biomass and the blanching water after blanching. Fucose represents the total fucoidan content. Glucose represents the total laminaran content of the blanching water but includes cellulose for the fresh biomass. Values are given as average ± std dev (*n* = 3).

	Content in the Fresh Biomass (% of dw)		
	Fucose	Glucose	Mannitol
Sugar kelp	0.93 ± 0.18	3.6 ± 1.0	7.7 ± 6.3
Winged kelp	1.02 ± 0.07	2.0 ± 0.2	2.7 ± 0.1
	**Content in the Blanching Water (mg L^−1^)**		
	**Fucose**	**Glucose**	**Mannitol**
Sugar kelp			
Standard	26.6 ± 2.6	1.88 ± 0.61	140 ± 6
45 °C, 30 s	6.59 ± 0.69	0.926 ± 0.123	11.9 ± 9.5
Cut	90.5 ± 6.7	2.68 ± 0.81	42.9 ± 29.2
SW, SW	0.933 ± 0.077	0.337 ± 0.089	9.32 ± 1.31
Rep 5	128 ± 81	48.5 ± 61.3	764 ± 644
Rep 10	296 ± 127	155 ± 127	2010 ± 1160
Winged kelp			
Standard	6.37 ± 0.51 *	0.666 ± 0.002 *	112 ± 0 *

* Average of two replicates with variation range.

## Data Availability

The original contributions presented in this study are included in the article/Supplementary Materials. Further inquiries can be directed to the corresponding author.

## Supplementary Materials

The following supporting information can be downloaded at: https://www.mdpi.com/article/10.3390/foods14234113/s1, Supplementary Tables, Full dataset in Excel, and Supplementary Figures, “Supplementary Figures.pdf”.
