# Supplementary material for: Blanching of Two Commercial Norwegian Brown Algae for Reduction of Iodine and Other Compounds of Importance for Food Safety and Quality
Source: Foods. 2025 Dec 1;14(23):4113. doi: 10.3390/foods14234113 (PMC12691785; doi:10.3390/foods14234113)
Supplement: Supplementary file 1 [file foods-14-04113-s001.zip › Supplementary Figures.pdf]

Sletta *et al*: Blanching of two commercial Norwegian brown algae for reduction of iodine and other compounds of importance for food safety and quality

Supplementary figures

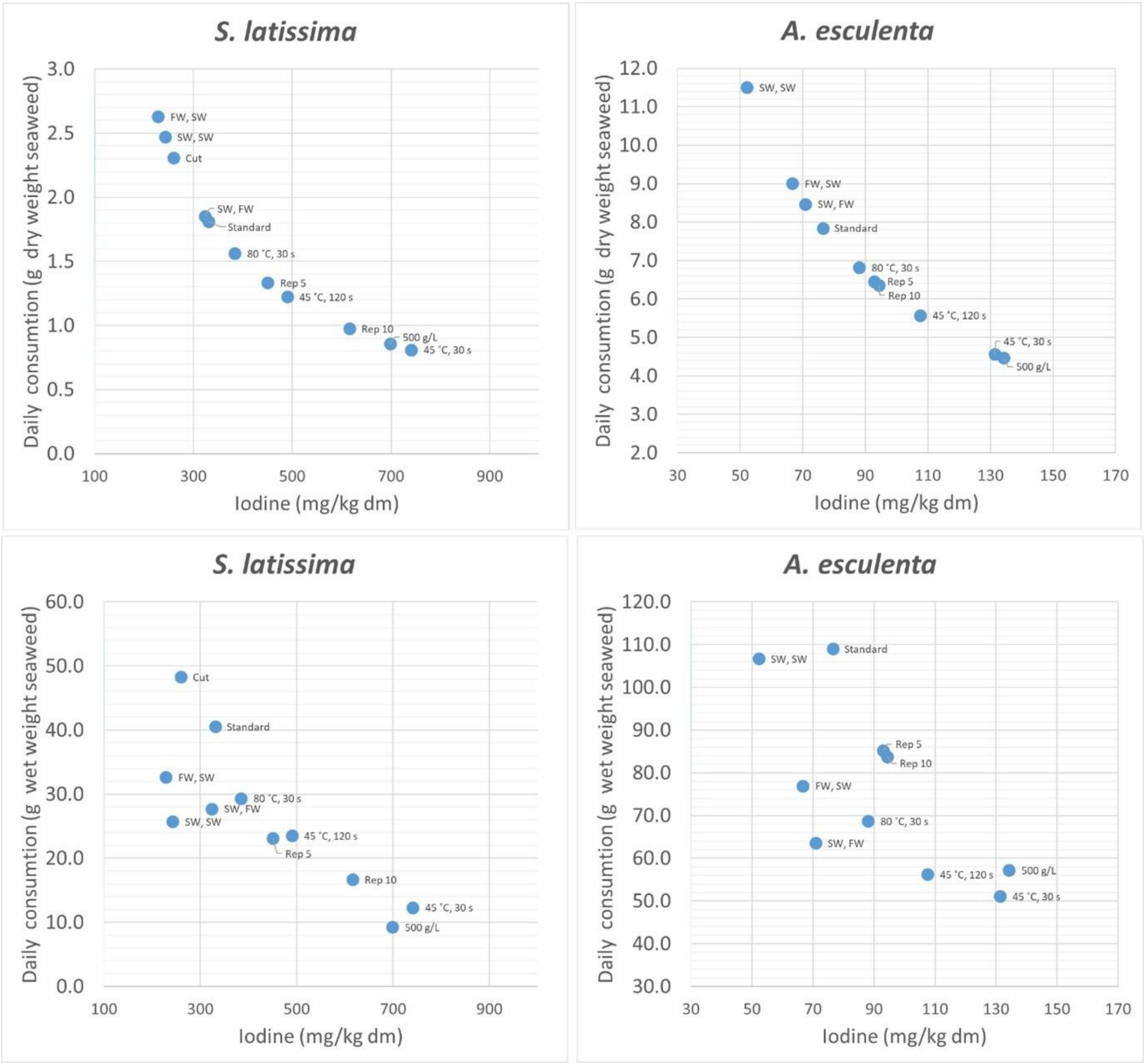

Figure S1: Acceptable daily consumption of biomass blanched at the indicated conditions, with the actual measured iodine concentrations as X-axis..

Sugar kelp (*Saccharina latissima*)

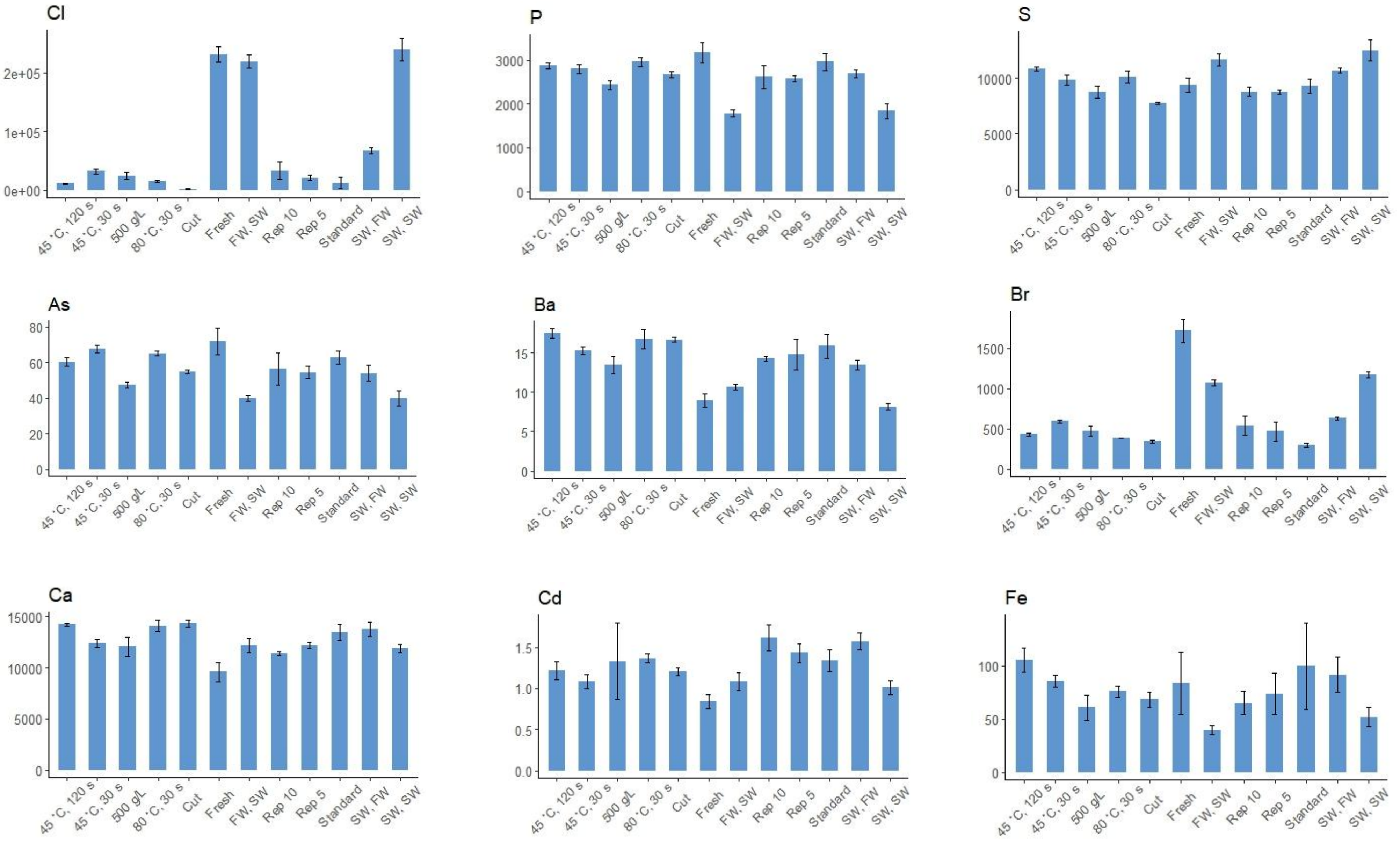

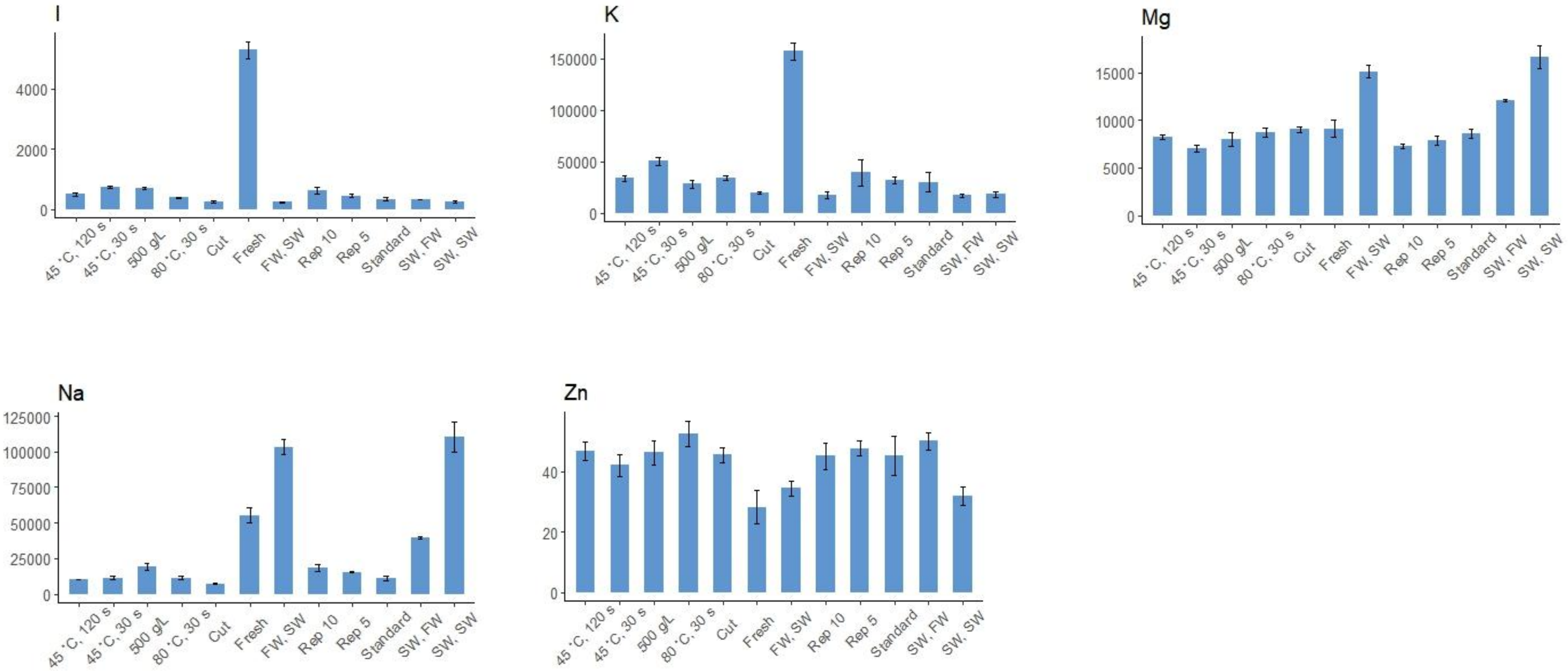
I
0
2000
4000
K
0
50000
100000
150000
Mg
0
5000
10000
15000
Na
0
25000
50000
75000
100000
125000
Zn
0
20
40
45 °C, 120 s
45 °C, 30 s
500 g/L
80 °C, 30 s
Cut
Fresh
FW, SW
Rep 10
Rep 5
Standard
SW, FW
SW, SW

Winged kelp (*Alaria esculenta*)

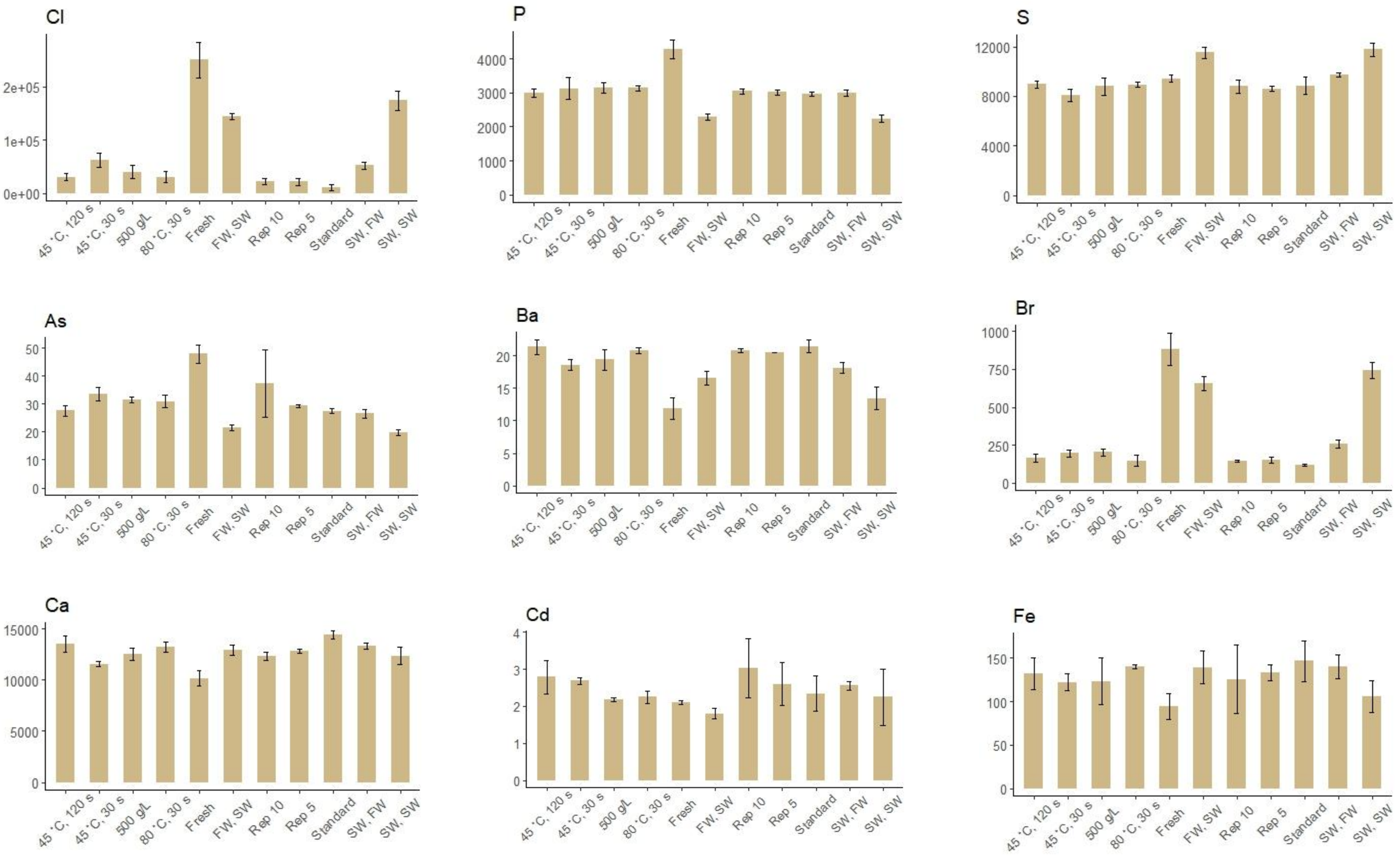

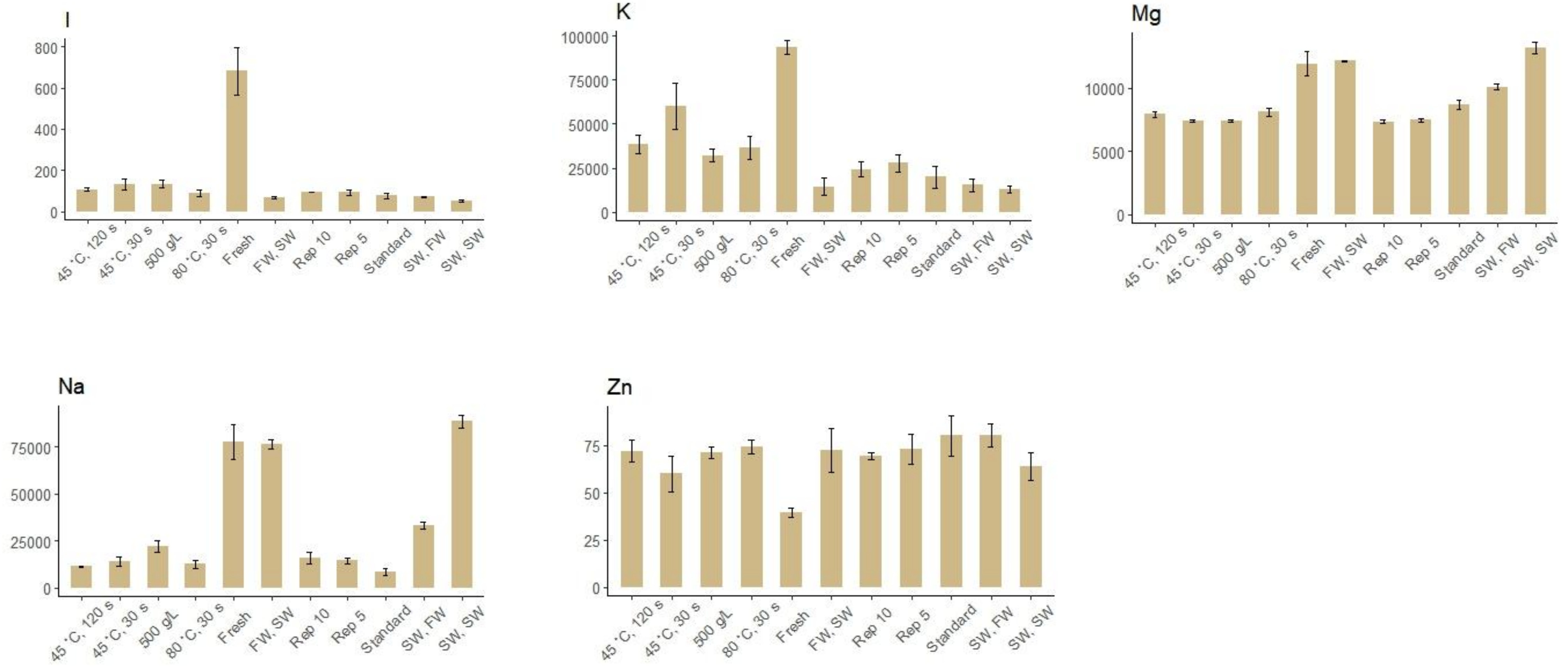

Figure S2: Mineral content [mg/kg dw] in fresh and blanched biomass.
In addition to the 14 minerals presented, Co and Se were determined but were below the respective detection limits of 0.25 and 2.5 mg/kg dw.

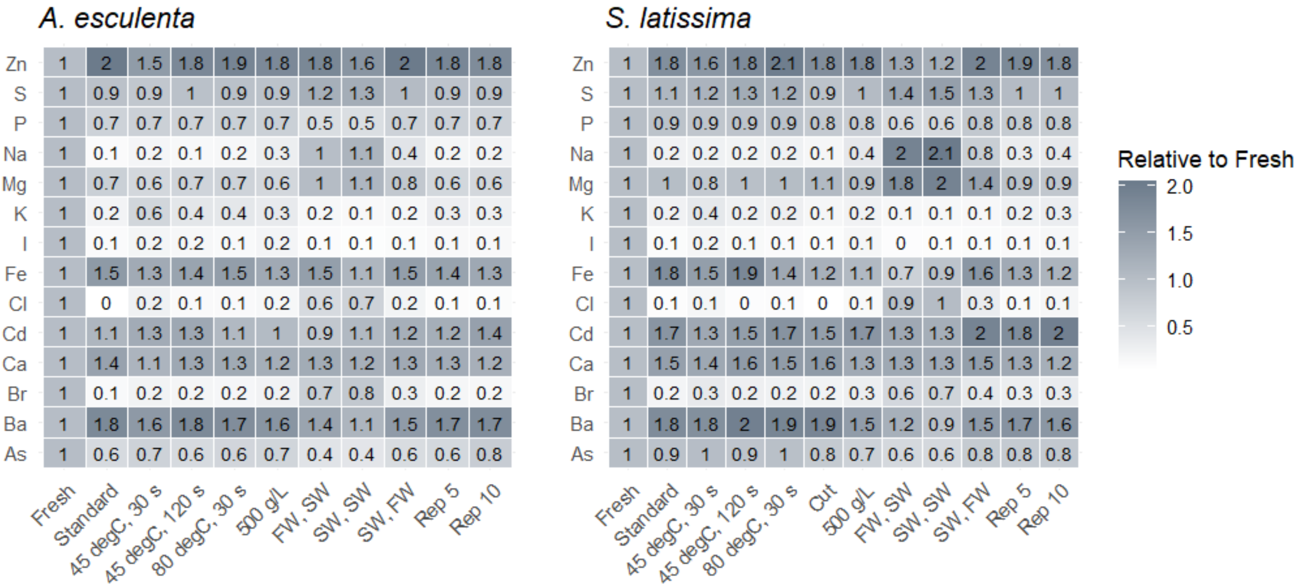

Figure S3: Changes in mineral content after blanching at the different conditions, relatively to fresh biomass.

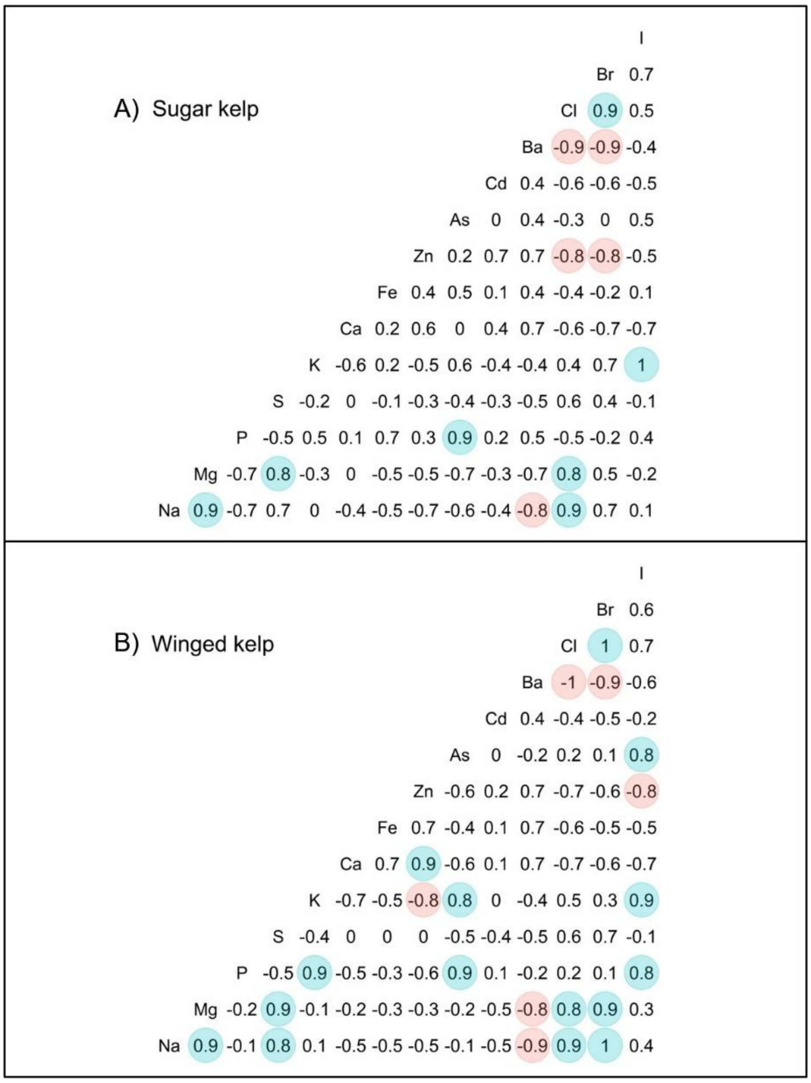

Figure S4: Pearson corelation coefficients between minerals remaining in the biomass after blanching, including all blanching conditions. The strongest correlations are highlighted in red (negative correlation) and blue (positive correlation).
